# Impact of Nitrous Oxide on Neuropsychiatric Impairment in Adolescents: A Scoping Review

**DOI:** 10.7759/cureus.89448

**Published:** 2025-08-05

**Authors:** Vanessa C Cornelio, Gary Lail, Kimberly Go, Kiran Kaur, Piotr Slowik

**Affiliations:** 1 Psychiatry, St. John's Episcopal Hospital, Far Rockaway, USA; 2 Gastroenterology and Hepatology, St. John's Episcopal Hospital, Far Rockaway, USA; 3 Anatomical Sciences, William Carey University College of Osteopathic Medicine, Hattiesburg, USA; 4 Internal Medicine, American University of the Caribbean School of Medicine, Cupecoy, SXM

**Keywords:** addiction medicine, inhalant use among adolescents, inhalant use disorder, longterm complication of nitrous oxide, nitrous oxide toxicity, treatment for nitrous oxide

## Abstract

Nitrous oxide (N₂O) misuse represents a growing public health issue, particularly among adolescents and young adults, who use it recreationally. This scoping review investigates recent and foundational studies on the long-term neuropsychiatric effects associated with prolonged and frequent use of this volatile gas. Various clinical databases and journal archives, such as those of the Journal of Addiction Medicine, Journal of Child Psychiatry, Journal of Anesthesiology, Science Direct, and National Survey of Drug Use and Health, were queried for clinical trials, case reports, and public health surveys. Emphasis was placed on studies exploring the neurobiological mechanisms, epidemiological trends, clinical manifestations, and treatment strategies associated with chronic N₂O exposure. The search revealed epidemiological factors like low socioeconomic status, peer influence, and concurrent use of alcohol or cannabis increased the likelihood of N₂O misuse. Biomechanically, N₂O acts via N-methyl-D-aspartate receptor (NMDA) receptor antagonism and endogenous opioid release, altering neurotransmitter dynamics and leading to potential dependence. Chronic use disrupts vitamin B12 metabolism by oxidizing methylcobalamin, impairing myelin production,n and leading to subacute combined degeneration of the spinal cord. Neuroimaging studies reveal deactivation of the hippocampus and visual cortices, aligning with observed memory and sensory processing deficits. Psychiatric complications such as depression, delusions, and agitation are frequently reported in adolescent users. Early intervention through cessation, vitamin B12 supplementation, and emerging pharmacologic agents such as naltrexone and aripiprazole can mitigate adverse outcomes. Public health initiatives such as educational campaigns, legislative control, and school-based prevention programs are critical to curbing ongoing misuse. Despite increasing attention, significant gaps remain in understanding long-term outcomes, effective treatments, and global epidemiological patterns. Further research is essential to develop evidence-based clinical guidelines and preventative strategies tailored to this at-risk population.

## Introduction and background

Nitrous oxide (N_2_O) is a colorless, non-irritating gas with a sweet aroma that has therapeutic roles in alleviating pain and anxiety in medical and dental procedures, as well as in the management of treatment-resistant depression. There are multiple purported mechanisms by which N_2_O exerts its analgesic effect, including via activation of the endogenous opioid system and induction of a preventative blockade of pain hypersensitivity by interrupting nociceptive inputs [1]. To demonstrate the efficacy of N_2_O in pain reduction, a randomized study published in 2019 indicated a statistically significant decrease in pain scores from 6.0 to 3.4 within five minutes of continuous inhalation [2]. Its role in treatment-resistant depression has been demonstrated in a double-blinded placebo-controlled crossover trial comprising 20 patients, where significant improvement in depressive symptoms was observed after two hours and sustained 24 hours post-treatment [3].

In addition to its therapeutic uses, N_2_O is also an up-trending recreational drug of choice among the adolescent and young adult population due to its psychedelic and euphoric effects [4]. According to the World Health Organization and the National Drug Survey, adolescents are in the age group of 12-17 years, and young adults are in the age group of 18-25 years [5]. Its misuse plausibly arises from the resulting emotional state of relaxation, dissociation, increased somnolence, and occasional hallucinations [6]. Furthermore, N_2_O produces physiological and psychological effects within seconds after inhalation, with peak impact at approximately one minute and a complete dissolution of these effects at three to five minutes [7]. Due to this quick inhalant experience, many individuals inhale N_2_O several times to prolong the intoxication, leading to long-term adverse effects on the neuropsychiatric development of adolescents. 

It is known that adolescents misusing N_2_O often present with a range of neuropsychiatric symptoms, most commonly auditory hallucinations and persecutory delusions. With long-term use, adolescents can experience agitation, depression, and personality changes [8]. There have also been reports of acute cognitive decline, such as memory impairment and confusion, which are noted to be early manifestations of repetitive use [9]. The National Institute on Drug Abuse (NIDA) highlights that chronic exposure can have a severe impact on the brain’s white matter, affecting essential functions like movement, vision, and cognition [10]. The study indicates that early exposure to inhalant use increases the risk of more severe substance use disorder, especially if use is started prior to the age of 18. This, combined with the stable, albeit minimal, uptrend in the use of N_2_O among adolescents, demonstrates the need to understand the biochemical mechanisms, as well as both the psychiatric and physiological manifestations, to aid clinicians in prompt recognition and treatment of this disorder.

Thus, the purpose of this scoping review was to compile existing literature that focuses on the long-term effects of N_2_O among adolescents. With the increasing prevalence of N_2_O misuse among adolescents, there is an immediate need for further investigation in order to protect the neurological and psychological well-being of this vulnerable subset of the population. 

Historical context and usage trends

Overview of N_2_O's History and Applications 

N_2_O is one of the oldest pharmaceutical drugs that is still used in clinical practice. Originally synthesized by Priestley in 1772, this volatile gas was first reported for its medical use by American dentist Horace Wells for a dental extraction [11]. Since its discovery and debut for analgesic and anesthetic properties in 1844, N_2_O pressure cylinders have been a staple for dental and minimally invasive medical procedures in the field of obstetrics, pediatrics, and chronic pain therapy, to safely administer the inhalant to patients [12]. In recent years, N_2_O has also been shown to be efficacious in treating refractory depression. Patients who received a mean duration of N_2_O treatment of 55.6 minutes at a median inspiratory concentration of 44% report a sustained improvement in mood and associated symptoms, days after treatment [3,13]. 

Recreational use of N_2_O has become increasingly popular over the decades due to its ability to induce dissociative and hallucinogenic effects of depersonalization, derealization, euphoria, lightheadedness, and auditory distortion. It is now one of the common inhalants used by adolescents and young adults at festivals and parties, typically going by its street names such as “whippets”, “whippits”, or “hippy crack”. This gas is usually inhaled through a balloon, bulb, or N_2_O canister in a process known as “nagging” [14]. This increase in use is found to be more prevalent among adolescents in rural or suburban areas, although misuse is documented in urban demographics as well. 

An investigative study conducted in 2022 by Inquimbert et al. probed into the factors contributing to the recreational use of N_2_O among healthy individuals at the Montpellier University in France [15]. The sample size consisted of 593 students with a mean age of 22.3 years; 68.6% of this sample population were female. The study observed that older male students who were completing their medical or dental studies and were part of a student corporation had significantly higher usage. Harmful habits such as tobacco, alcohol, or cannabis consumption were significantly associated with N_2_O use. It is also interesting to note that nearly one-quarter of N_2_O users were not aware of their misuse. Desirable factors such as euphoria, impaired vision, or fun were significantly associated with an increased frequency and dosage of N2O use [15].

Statistics on Adolescent Use and Demographic Factors

There has been an increase in the prevalence of N_2_O users in the Western world since the early 2000s. A 2014 global drug survey involving 17 countries and 74,684 participants concluded that N_2_O has become increasingly popular. The United States reported a 29.4% use of this gas. While the timeline remains unclear on the origin of N_2_O as a substance of misuse, the earliest known case report on the euphorigenic effects of N_2_O was in 1961 [14]. The lifetime prevalence of this inhalant misuse among adolescents and young adults ranges between 2% and 15.8% [4,16].

The National Survey on Drug Use and Health (NSDUH) indicated that the lifetime use of N_2_O across populations was 4.9% in 2004 (ages 12-17: 1.8%; ages 18-25: 10.2%; ages 26 and over: 4.4%) [17]. The most recent data in 2023 reported a lifetime N_2_O use of 4.9% total (ages 12-17: 0.4%; ages 18-25: 3%; ages 26 and over: 5.3%) [18]. The data suggests a fairly stable rate of usage, with an increase in use observed among young adults aged 18-25 years.

A cross-sectional study conducted by van den Toren et al. investigated the socio-demographic characteristics associated with recreational N_2_O use in a sample population of 555 adolescents [19]. The study concluded that students with a lower socioeconomic background, lower educational levels with externalized issues such as familial conflict or peer pressure, and frequent alcohol binging, had a statistically significant increase in N_2_O use. This risk was observed in 15.6% of students from the study sample population [19].

## Review

Mechanisms of action

N_2_O is rapidly absorbed via inhalation. Due to its relative insolubility in blood and adipose tissue, the vapor has a rapid onset and offset of effects [20]. N_2_O causes the activation of opioid receptors in the brainstem, which provides its analgesic effects by inhibiting gamma-aminobutyric acid-releasing neurons while simultaneously activating descending noradrenergic pathways to inhibit pain [21]. N_2_O is also able to release endogenous opioids directly from the peri-aqueductal grey area of the midbrain, causing activation of the μ-opioid and κ-opioid receptors, thereby potentially contributing to dependence and misuse. An alternative mechanism of addiction could be the blockade of NMDA-type glutamate receptors, which aids in inducing dopamine disinhibition by GABAergic neurons in the ventral tegmental area and the nucleus accumbens [21]. As a result, there is a dopamine “burst firing” that increases the likelihood of addiction. There is a continuation in brain development during adolescence and the young adult period. During this time, there is heightened plasticity in the NMDA receptors that are influenced by experience-dependent learning mechanisms [22]. This increase in the NMDA receptor capacity results in the adolescent and young adult brain being more susceptible to thrill-seeking and addictive behavior. 

The influence of N_2_O on changes in neurological pathways that potentiate psychosis remains a point of debate in the scientific world. Hutto speculates that psychosis results from increased synthesis of tetrahydrobiopterin (BH4), affecting the rate of synthesis of the monoamines (dopamine, norepinephrine, and serotonin) [23]. Others have reported that N_2_O leads to psychotic symptoms via cerebral anoxia, methemoglobinemia, low arterial pO_2_, and acidosis. A mini review conducted by Fluegge demonstrates that exposure to N_2_O, even at nontoxic doses, may influence central neurotransmission and target neurotransmitters in pathways known to modulate neurodevelopment, such as the glutamatergic, opioidergic, cholinergic, and dopaminergic pathways [24]. A review conducted by Patel et al. highlights the vulnerability of adolescent neurodevelopment to psychosis [25]. Their review focused on important biological factors, such as accelerated neuronal pruning that alters cortical thickness and blunted myelination that increases the risk of psychosis among adolescents. These processes are dramatically affected by nitrous oxide misuse, affecting the synthesis and processing of BH4. 

Many case studies have indicated a low level of vitamin B12 in N_2_O users. It has been postulated that N_2_O inactivates methylcobalamin by oxidizing the cobalt ion from 1+ to 3+ valence states [21,23]. By doing so, methionine synthase cannot convert the amino acid homocysteine into methionine, thereby causing a decrease in myelin production. There is also an impaired ability to convert 5-methyltetrahydrofolate to tetrahydrofolate, negatively affecting the production of DNA synthesis. This can enhance myelin inflammation, causing damage to the posterior columns and the corticospinal tract of the spinal cord.

Neuropsychiatric effects of nitrous oxide

Human behavioral studies have depicted impairments in psychomotor function and cognitive performance with the chronic use of N_2_O. A study conducted by Gylai et al. using positron emission tomography (PET) and regional cerebral blood flow indicated areas of the brain that were affected by N_2_O [26]. Their study used eight volunteers to demonstrate activation of the anterior cingulate cortex, which is known to mediate psychomotor and cognitive processes, and also demonstrates deactivation of the posterior cingulate, hippocampus, parahippocampal gyrus, and visual hemispheres in both cerebral hemispheres. The latter regions are known to mediate learning and memory. Further study of these areas may hence provide a foundation for the changes in behavioral responses to N_2_O use.

A case report published in 2023 followed a 22-year-old male patient with a history of inhaling six 615-gram N_2_O cylinders daily for two weeks [27]. It was observed that the patient suffered from rapidly progressing paresthesia, gait difficulties, short-term and long-term memory loss, and extrapyramidal symptoms such as dystonic posturing and athetosis. Neuroimaging demonstrated subacute combined degeneration of the spine. Hematological labs indicated low serum cobalamin and elevated levels of homocysteine. The patient's symptoms were reversible with parenteral hydroxocobalamin.

Neurological abnormalities are hence the most prominent features of chronic N_2_O use. As seen in the case report by Lindeman et al., subacute combined degeneration is the most common disorder [27]. Other common disorders with chronic use include peripheral neuropathy, such as gait disturbance, bilateral paresthesia, hyporeflexia, and encephalopathy, such as behavioral changes, delusions, hallucinations, and paranoia. Acute intoxication usually presents with altered cognition, hypoxemia, asphyxia, and cold-related injury to the mouth and hands [28]. Thus, N_2_O toxicity should be considered in the differential diagnosis for all patients with signs and symptoms of peripheral neuropathy and encephalopathy. A detailed history of chronic N_2_O exposure, vitamin B12 levels, and functional MRI is necessary to reach an accurate diagnosis. A low vitamin B12 level is observed in 54-72% of patients with a history of chronic use and insidious neurological symptoms [28].

Interventions and treatment strategies

The primary treatment for N_2_O misuse is cessation and vitamin B12 supplementation. A case report published in 2023 indicated that naltrexone may be a plausible pharmacological intervention that can be used to help in cessation [29]. Aripiprazole has also been shown to have positive effects in the cessation of inhalant use disorder and may be considered as an alternative for the treatment of N_2_O addiction [30].

Given the low cost and easy access to N_2_O, clinicians need to be aware of this recreational drug used by the younger population. Awareness and counseling about the use of cartridges should be emphasized to minimize misuse and reduce both the acute and chronic effects [28]. Educational campaigns, age limit restrictions, and school-based prevention programs are effective strategies to curb misuse [31]. In addition to knowing the management of misuse, it is also important to have early intervention, as adolescents are more susceptible to the neurotoxic effects of N_2_O misuse.

## Conclusions

Despite growing investigative research on N₂O misuse, significant gaps remain in the literature, especially regarding the long-term consequences of use among adolescents. This review draws attention to several key impairments associated with chronic N_2_O exposure, which include cognitive deficits, psychosis, mood disturbances, emotional dysregulation, and behavioral changes. These impairments are detrimental during adolescence, a critical period of neurodevelopment characterized by heightened vulnerability to psychiatric disorders and long-term functional impairment.

Variability in study designs, outcome measures, and populations made it impractical for a meaningful quantitative synthesis, and due to these limitations, we employed a narrative approach. This method enabled us to summarize and contextualize the findings in a clinically relevant manner. Future research should aim to identify potential biomarkers and neuroimaging patterns to better characterize the underlying pathology of N₂O-related neuropsychiatric impairment. Studies are also needed to evaluate the efficacy of various therapeutic interventions in addressing both neurological and psychological sequelae. Furthermore, cross-cultural investigations are warranted to understand the global epidemiology of N₂O misuse and assess the effectiveness of different prevention strategies.
